# Development of a Non-Invasive On-Chip Interconnect Health Sensing Method Based on Bit Error Rates

**DOI:** 10.3390/s18103234

**Published:** 2018-09-26

**Authors:** Insun Shin, Kyoungmin Koo, Daeil Kwon

**Affiliations:** School of Mechanical, Aerospace, and Nuclear Engineering, Ulsan National Institute of Science and Technology, Ulsan 44919, Korea; ishin@unist.ac.kr (I.S.); kkoo@unist.ac.kr (K.K.)

**Keywords:** bit error rates, interconnect, prognostics, diagnosis, health management

## Abstract

Electronic products and systems are widely used in industrial network systems, control devices, and data acquisition devices across many industry sectors. Failures of such electronic systems might lead to unexpected downtime, loss of productivity, additional work for repairs, and delay in product and service development. Thus, developing an appropriate sensing technique is necessary, because it is the first step in system fault diagnosis and prognosis. Many sensing techniques often require external and additional sensing devices, which might disturb system operation and consequently increase operating costs. In this study, we present an on-chip health sensing method for non-destructive and non-invasive interconnect degradation detection. Bit error rate (BER), which represents data integrity during digital signal transmission, was selected to sense interconnect health without connecting external sensing devices. To verify the health sensing performance, corrosion tests were conducted with in situ monitoring of the BER and direct current (DC) resistance. The eye size, extracted from the BER measurement, showed the highest separation between the intact and failed interconnect, as well as a gradual transition, compared with abrupt changes in the DC resistance, during interconnect degradation. These experimental results demonstrate the potential of the proposed sensing method for on-chip interconnect health monitoring applications without disturbing system operation.

## 1. Introduction

Timely maintenance of systems and assets is critical for the prevention of loss in revenue owing to unexpected system downtime. Therefore, proactive maintenance strategies were developed that are optimized to increase the availability of products and systems. In particular, condition-based maintenance is a proactive maintenance strategy that involves monitoring the performance and environmental conditions of a target system. One of the key factors associated with proactive maintenance is the use of appropriate sensors to collect system health data for fault diagnosis and prognosis.

Many researchers conducted studies to develop system health monitoring methods using electrical parameters in the time or frequency domain. Gershman et al. [1] suggested a quantitative measurement tool based on the serial ohmic resistance changes at solder joints; the serial ohmic resistance in such cases was used to monitor crack propagation in the solder joints of a quad flat non-leads (QFN) package. Their experimental results showed that the serial ohmic resistance increased as a result of the crack growth. Ji et al. [2] proposed a condition monitoring method for insulated-gate bipolar transistor (IGBT) power modules based on voltage measurements. In particular, they designed an additional measurement circuit for the monitoring system to measure voltage. Ralph et al. [3] applied acoustic emission detection to detect damage in the solder interconnections between components and the printed circuit board. Wang et al. [4] used joint time–frequency domain reflectometry (JTFDR) to monitor incipient power cable faults; their results showed that the JTFDR time–frequency cross correlation peak increased during cable aging testing. Okoro et al. [5] used a vector network analyzer (VNA) to measure the S-parameters of a through-silicon via (TSV) stacked die, and showed the potential of high-frequency signal-based measurement for assessing the reliability of TSV stacked dies. Researchers also reported defect sensing methods using eddy current pulsed thermography [6,7] and acoustic micro imaging [8]. Zheng et al. [9] applied normal fiber Bragg grating (FBG) sensors to have real-time health monitoring of composite materials. Aryan et al. [10] reviewed the sensing devices, such as X-ray, scanning acoustic microscope, infrared (IR) camera sensor magnetic field, and optical vibration measurement, for integrated circuit (IC) package inspection. Nevertheless, these existing methods often require external and additional sensing devices, such as multimeters, oscilloscopes, and VNAs, which might disturb real-time system operation of these packages or devices, and eventually increase operating costs. Therefore, a non-destructive and non-invasive health monitoring method is required for fault diagnosis and prognosis that helps in reducing monitoring time and costs.

In this study, we present an on-chip health sensing method for non-destructive and non-invasive interconnect degradation detection based on digital signal characteristics; in particular, bit error rate (BER), defined as the ratio of the number of erroneously transmitted bits to the generated bits, was used to measure the physical degradation of an interconnect. Accelerated life tests on solder joints were conducted with in situ monitoring of the BER and DC resistance to verify the health sensing performance of the proposed method, as well as compare it with that of the DC resistance measurement. The advantage of the proposed method is that BER, which can be programmed and analyzed using a chip, does not require external and additional devices to monitor interconnect degradation.

The remainder of this paper is organized as follows: Section 2 introduces the concept of digital signal characteristics, and how BER is measured. Section 3 presents our proposed interconnect health sensing method based on BER. The experiment-based sensing performance validation of our proposed approach is discussed in Section 4. Finally, Section 5 summarizes and concludes the paper.

## 2. Digital Signal Characteristics

**Figure 1:**
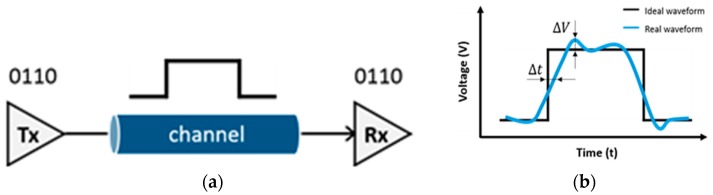
Digital signal transmission: (**a**) conceptual representation (**b**) ideal and real waveforms.

Electronic devices primarily operate based on digital signals, which are a series of discrete pulses, representing logical ones and zeroes. These digital signals have high network capacities owing to multiplexing, enabling multiple communication lines over a single communications channel. Thus, digital signals are transmitted through channels and interconnects in electronics. Figure 1a depicts a digital signal transmission system, which consists of a transmitter, receiver, and channel. Although digital signals are a sequence of discrete pulses, noise can affect ideal digital signals, as shown in Figure 1b. The deviation of a noisy signal from an ideal signal can be considered from two different aspects: timing deviation (Δ*t*) and amplitude deviation (Δ*V*) [11]. Timing deviation is defined as timing jitter, whereas amplitude deviation is defined as amplitude noise. In particular, timing jitters affect the performance of a transmission system at the edges of signal transitions, whereas amplitude noise affects the performance during the signal transitions themselves. Signal integrity (SI) is a set of digital signal characteristics affected by timing jitter and amplitude noise. SI analysis involves evaluating whether the signal reaches its receiver as scheduled without a loss in logic levels. Thus, a digital signal with good SI has fast transitions, valid logic levels, and accurate time placement [12]. SI analysis is widely used for electronic packages and assemblies to evaluate the electrical performances of their internal connections in the case of integrated circuits (ICs), printed circuit boards (PCBs), backplanes, and inter-system connections.

Thus, digital signals can serve as a means for detecting physical degradation of interconnect surfaces based on the skin effect [13,14], which is a phenomenon wherein high-frequency signals accumulate near the surface of a conductor. Yoon et al. [13] presented a diagnosis method for solder joints by monitoring eye parameters, which are sets of parameters obtained from SI analysis. Eye parameters were collected during solder joint degradation testing, and the observed jitters showed significant deviation with the degradation of the solder joints subjected to thermo-mechanical stress conditions. Furthermore, Lee et al. [14] developed a health monitoring system based on eye parameter measurement; in their study, the eye height extracted from the eye diagram showed gradual degradation under an accelerated life test of solder joints, and it was used for anomaly detection of the solder joints.

BER, which can also be defined as the number of error bits compared to the total number of bits received over a data communication channel, is affected by the physical degradation of a transmission channel. In digital transmission, error bits are generated when the incoming digital signals do not satisfy the bit decision criteria of a receiver owing to noise or damage to the transmission channel. BER is widely used to describe the overall performance of a communication channel based on the rate of error bits. Thus, in this study, we developed an on-chip monitoring method for non-destructive and non-invasive interconnect health sensing based on BER.

## 3. BER-Based Interconnect Health Sensing

**Figure 2:**
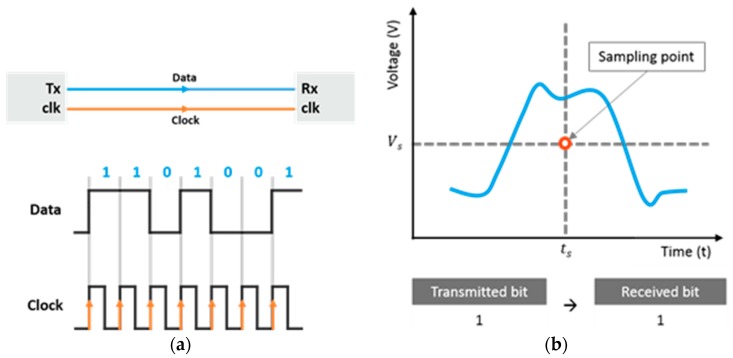
Signal sampling at a receiver. (**a**) data transmission of a synchronous bus (**b**) sampling point at a receiver.

Bits are determined based on a sampling point at the receiver. Figure 2a shows an example of data transmission on a synchronous bus. A receiver samples the transmitted data at a specific interval; the clock determines the sampling time $t_{s}$. At a sampling time, the receiver classifies the incoming bits as either 0 or 1 based on a threshold voltage $V_{s}$. Figure 2b shows a sampling point of a receiver and the manner in which the receiver determines the value of the bits. In particular, the receiver recognizes a bit as a logical one when the signal voltage is over a threshold voltage, or a logical zero when the signal voltage is lower than that threshold voltage.

**Figure 3:**
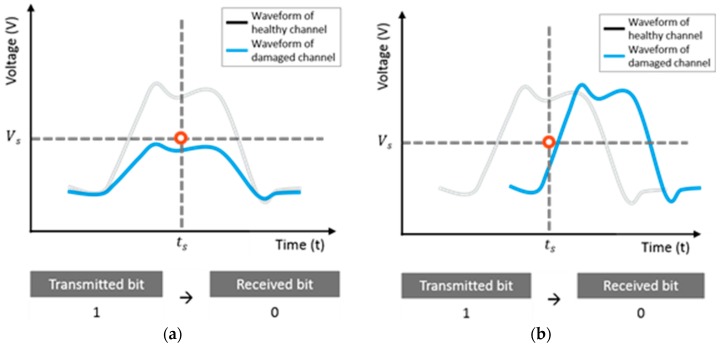
Bit error generation (**a**) by signal loss and (**b**) by timing noise.

In the presence of jitter and noise, the rising and falling edges of the signals may deviate along the time axis, and the voltage may fluctuate along the amplitude axis. Figure 3 shows examples of bit error generation due to jitter, noise, and signal loss. Owing to these issues, correct bit detection might not be achieved, resulting in a bit error, such as a logical one being detected as a logical zero, or vice versa. Furthermore, violations of sampling conditions can occur in the following three scenarios [11]: (1) the crossing time of the rising edge lags behind the sampling time, $t_{r}>t_{s}$; (2) the crossing time of the falling edge is ahead of the sampling time, $t_{f}<t_{s}$; and (3) the logical one voltage is below the sampling voltage, or $V_{1}<V_{s}$, where $t_{r}$ is the rise time, $t_{f}$ is the fall time, and $V_{1}$ is the voltage for the logical one.

**Figure 4:**
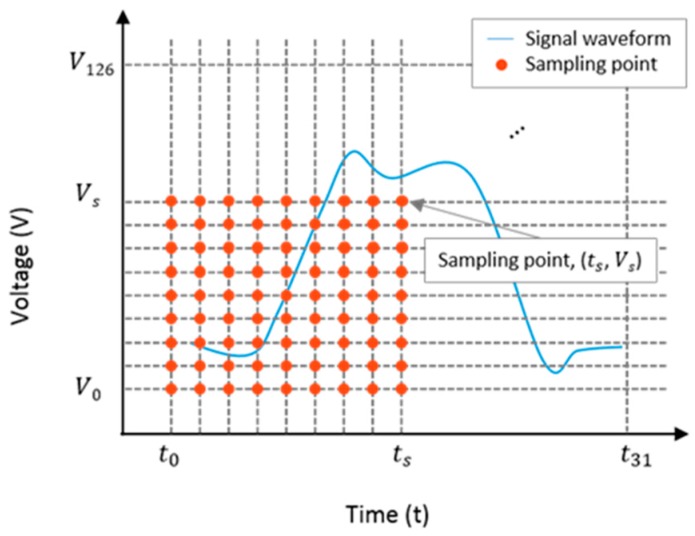
Bit error rate (BER) sampling points of the monitoring system.

**Figure 5:**
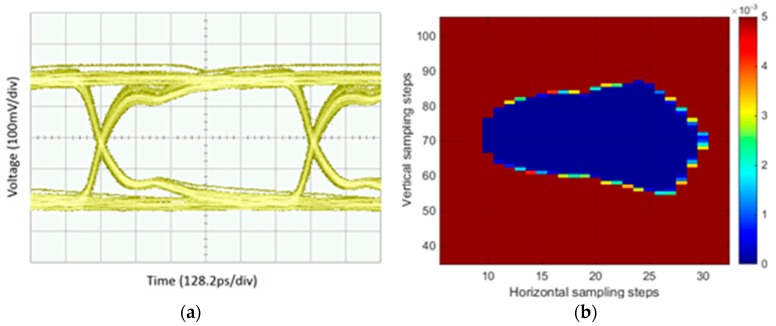
Digital signal waveform obtained using (**a**) an oscilloscope and (**b**) using BER.

BER-based interconnect health sensing was developed based on the bit detection mechanism. In order to monitor the degradation of digital signals, the sampling points were moved across the time interval, as well as the voltage interval, of a unit clock. Figure 4 illustrates 32 horizontal and 127 vertical sampling points of a unit clock. The sampling times moved from $t_{0}$ to $t_{31}$, and the threshold voltage moved from $V_{0}$ to $V_{126}$. Finally, the number of sampling points was 4064. The BER was measured at all the sampling points and the obtained values were arranged in the form of a matrix represented as follows:

(1) $$\left[\begin{array}{ccc} \begin{array}{ccc} BER_{0,126} & BER_{1,126} & BER_{2,126} \end{array} & \cdots & BER_{31,126}\\ \vdots & \ddots & \vdots \\ \begin{array}{ccc} BER_{0,2} & BER_{1,2} & BER_{2,2}\\ BER_{0,1} & BER_{1,1} & BER_{2,1}\\ BER_{0,0} & BER_{1,0} & BER_{2,0} \end{array} & \cdots & \begin{array}{c} BER_{31,2}\\ BER_{31,1}\\ BER_{31,0} \end{array} \end{array}\right],$$

where $BER_{a,b}$ is the BER at a sampling point when the sampling time is $t_{a}$ and the threshold voltage is $V_{b}$. A digital signal waveform measured using an eye diagram and using the BER of a healthy solder joint showed a good match; this is shown in Figure 5.

**Figure 6:**
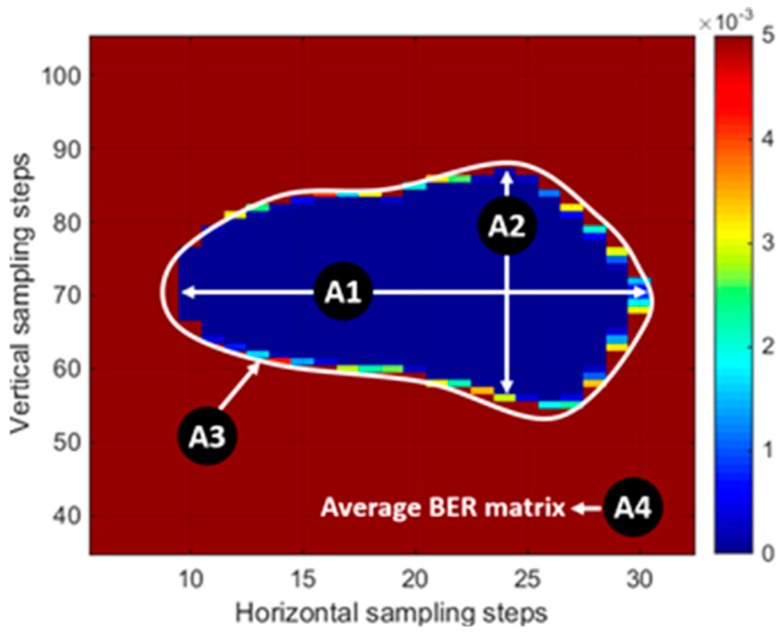
Feature sets based on the BER matrix.

Figure 6 and Table 1 show the feature description obtained from a BER matrix. In order to evaluate whether the extracted features are suitable for detecting solder joint failures, the distribution of each feature was used to assess the solder joint states. The mean and variance of the feature distributions from both normal and abnormal states were used for feature selection to calculate Fisher’s linear discriminant, which is a distance index between classes, the features to be selected in this study. Fisher [15] defined the separation index, S, between two distributions as the ratio of the variance between the classes to the variance within these classes:

(2) $$S=\frac{\sigma_{between}^{2}}{\sigma_{within}^{2}}=\frac{{\left(\vec{\omega }\cdot {\vec{\mu }}_{1}-\vec{\omega }\cdot {\vec{\mu }}_{0}\right)}^{2}}{{\vec{\omega }}^{T}\sigma_{1}^{2}\vec{\omega }+{\vec{\omega }}^{T}\sigma_{0}^{2}\vec{\omega }}=\frac{{\left(\vec{\omega }\cdot \left({\vec{\mu }}_{1}-{\vec{\mu }}_{0}\right)\right)}^{2}}{{\vec{\omega }}^{T}\left(\sigma_{0}^{2}+\sigma_{1}^{2}\right)\vec{\omega }},$$

where ${\vec{\mu }}_{0},\;{\vec{\mu }}_{1}$ and $\sigma_{0}^{2},\;\sigma_{1}^{2}$ are the means and covariances of the two distributions, respectively; and vector $\vec{x}$ represents the observations, while $\vec{\omega }$ is the normal to the discriminant hyperplane. In this study, the feature with a high separation index was used to detect faults in solder joints.

**Figure 7:**
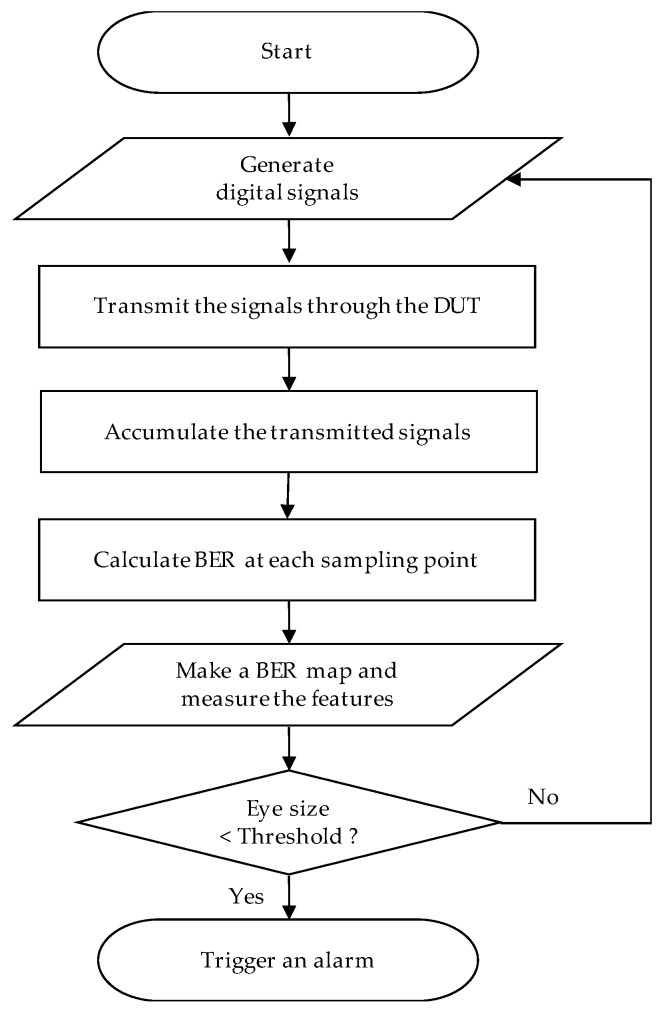
On-chip health sensing flowchart using BER.

Figure 7 shows the on-chip health sensing flowchart using BER as a summary. It starts with generating digital signals, transmitting them through the device under test (DUT), and accumulate the transmitted signals to create a BER map. The features, including eye size, are calculated from the BER map, and the health status is determined by comparing the feature with the pre-determined threshold.

## 4. Experimental Verification

**Figure 8:**
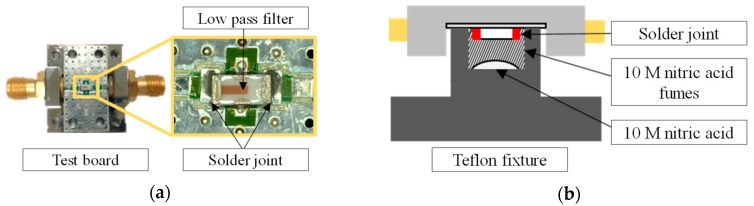
Test board and test fixture used in the accelerated life test. (**a**) test board (**b**) schematic of the test environment.

**Figure 9:**
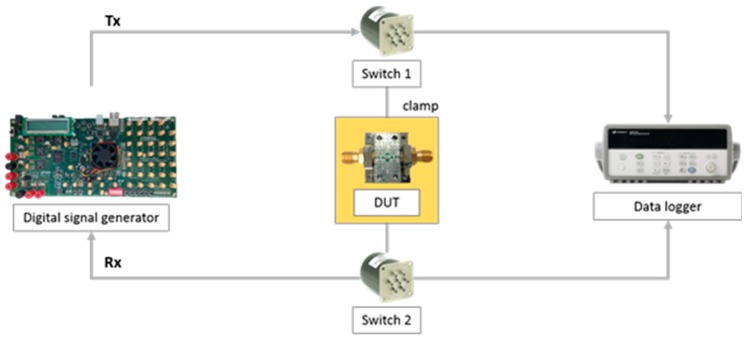
Test setup.

Accelerated life testing was conducted to demonstrate whether BER measurements can be used to detect solder joint degradation prior to interconnect failures. Corrosion tests induced failures of solder joints while collecting the degradation data using the on-chip health sensing method. In particular, the solder joints were exposed to fumes of 10 M nitric acid solution. The device under test (DUT) used in the experiment was a controlled impedance test board on which a low-pass filter component was soldered to transmit digital signals; this is shown in Figure 8a. SAC305 alloy was used to solder the low-pass filter to the test board. A Teflon fixture was produced in order to expose only the solder joints to the fumes and not the other interconnect and ground. Figure 8b shows the schematic arrangement of the DUT. Both BER and DC resistance were alternately monitored during the accelerated life test in order to examine their respective failure sensing capabilities. Figure 9 shows the schematic arrangement of the accelerated life test set-up, which consists of the DUT, digital signal generator, data logger, and switches. An Altera Stratix V GX was used to generate pseudorandom binary sequence (PRBS7) digital signals at a rate of 1.25 Gbps. The digital signals were generated and transmitted to the DUT through a switch. During the test, the four-wire DC resistance was monitored using a Keysight 34970A datalogger. The BER and the DC resistance were measured every 2 min alternately.

**Figure 10:**
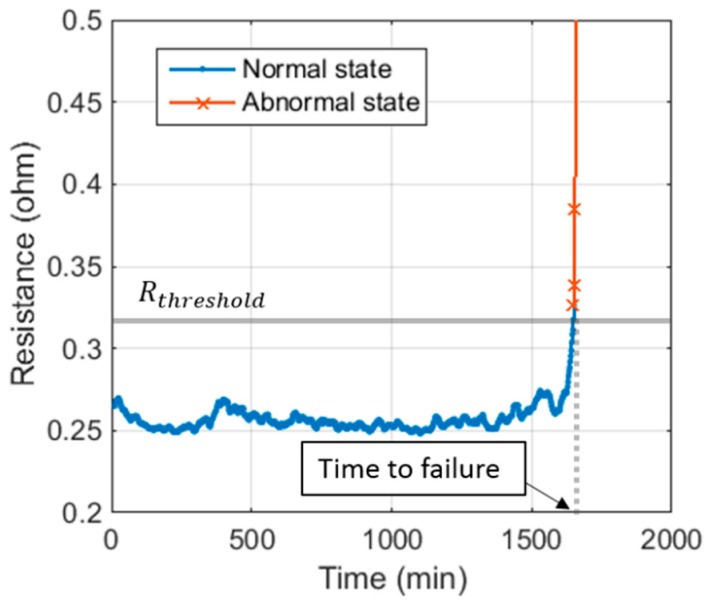
Direct current (DC) resistance measurement during corrosion testing (Test #1).

Figure 10 shows the DC resistance and time to failure from Test #1. The time to failure for the solder joints was recorded at 1650 min based on the international standards IPC-9701 [16] and JEDEC-9702 [17]. The initial resistance was observed to be around 0.27 Ω on average, while the threshold resistance, which showed an increase of 20% from the initial resistance, was 0.32 Ω. Normal state was defined to be the test duration before the time to failure, whereas an abnormal state was defined to be the one after the time to failure until the conclusion of the test, indicating a complete separation of the solder joint. The number of data points in the normal and abnormal states was 840 and 50, respectively.

**Figure 11:**
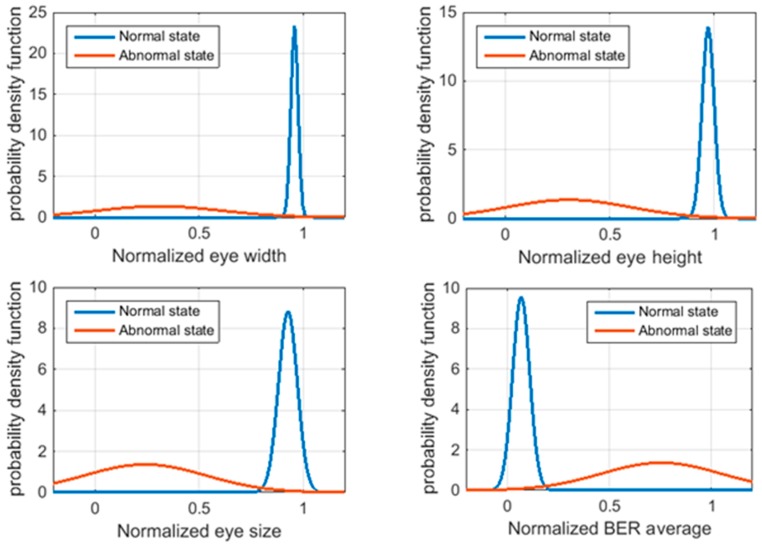
Probability density function of the features extracted from the BER matrix (Test #1).

Figure 11 shows the probability density functions of the four features extracted from the BER matrix in normal and abnormal states. Normal distribution was assumed for both normal and abnormal states. Eye width, eye height, and eye size indicated the normal states as one, and they decreased toward zero with the deterioration of solder joints. In contrast, a BER average of zero indicated as the normal state; the larger the BER average, the larger the number of bit errors in the BER matrix was. It was observed that the distributions of each state were sufficient to distinguish one from the other.

Multiple corrosion tests (test #1–#3) were performed under the same experimental conditions to ensure repeatability and reproducibility. Table 2 showed the separation analysis results of BER features. The separation index was calculated to select the feature that maximally separated means and narrow dispersions between the normal and abnormal states. The eye size showed the highest separation index out of all the four features; this could be attributed to the fact that the eye size contains more general geometric information about SI than the other features. In particular, the eye size indicates the size of the regions where the digital signal satisfies the error detection criteria. The eye width and eye height indicate the manner in which the digital signal passes in horizontal and vertical views, respectively. In this study, eye size was used for fault diagnosis.

**Figure 12:**
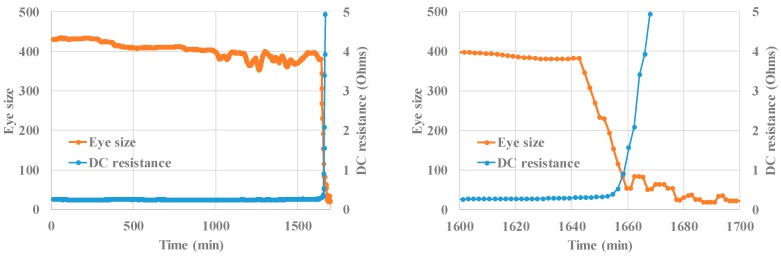
Eye size, DC resistance, and time to failure based on the feature set (Test #1).

**Figure 13:**
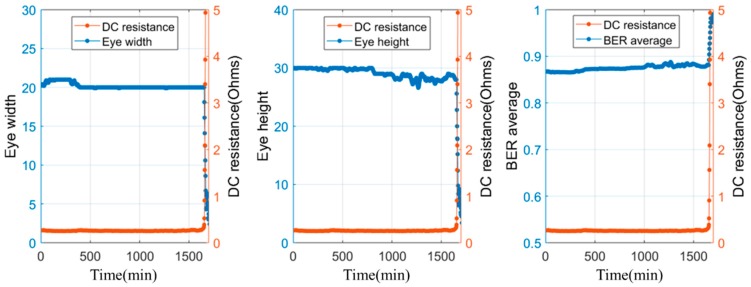
Eye width, eye height, BER average during solder joint corrosion (Test #1).

Figure 12 shows the eye size and DC resistance with their corresponding times to failure during corrosion testing. The DC resistance remained almost constant over most part of the test, and exhibited an abrupt increase near the time to failure; in this case, the time to failure was 1671 min. In contrast, the eye size gradually decreased during the test duration, and the time to failure, i.e., the time at which a 20% decrease was observed from the initial value by following the DC resistance criterion, was noted as 1680 min. After the time to failure, the eye size began to show a steeper decrease until the end of the test. Table 3 shows a summary of sensing results. Relative accuracy is defined to be the difference in the detected time to failure between BER (eye size) and DC resistance, divided by the detected time to failure using DC resistance. In the case of all the tests, the failure detection based on the eye size was close to the time of failure detected based on the DC resistance measurement; in particular, the sensing time difference was less than 0.6% of the total test time. Other BER features during corrosion testing are shown in Figure 13.

**Figure 14:**
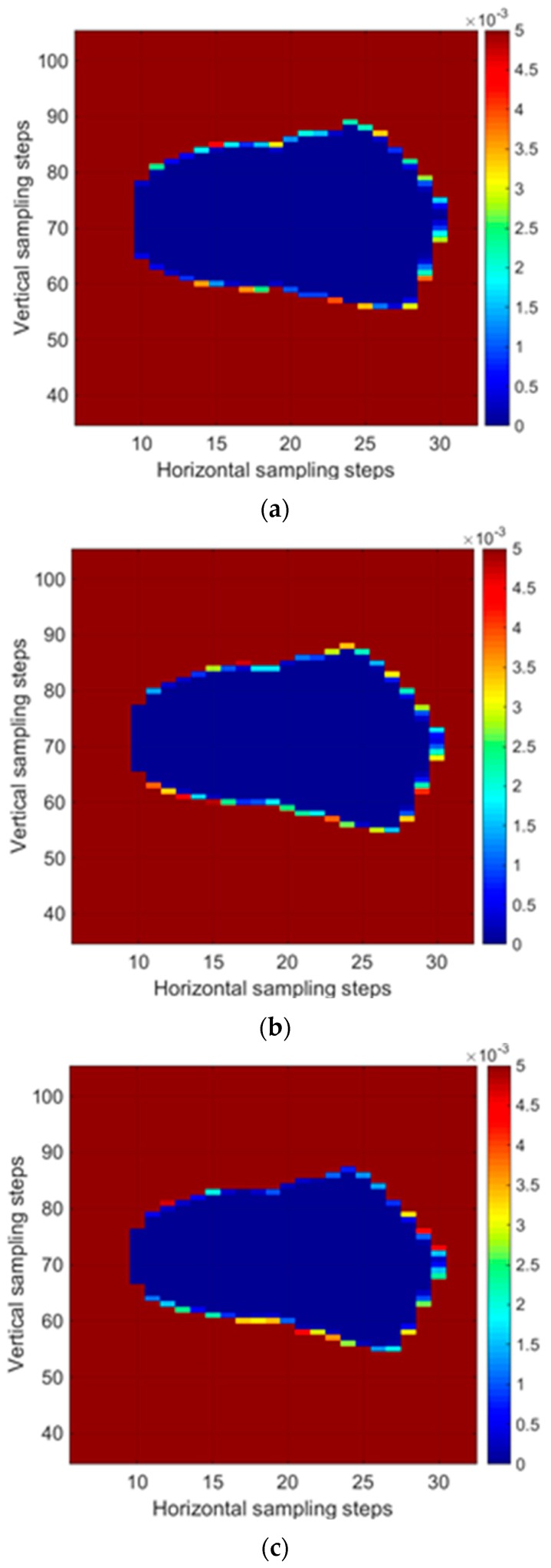

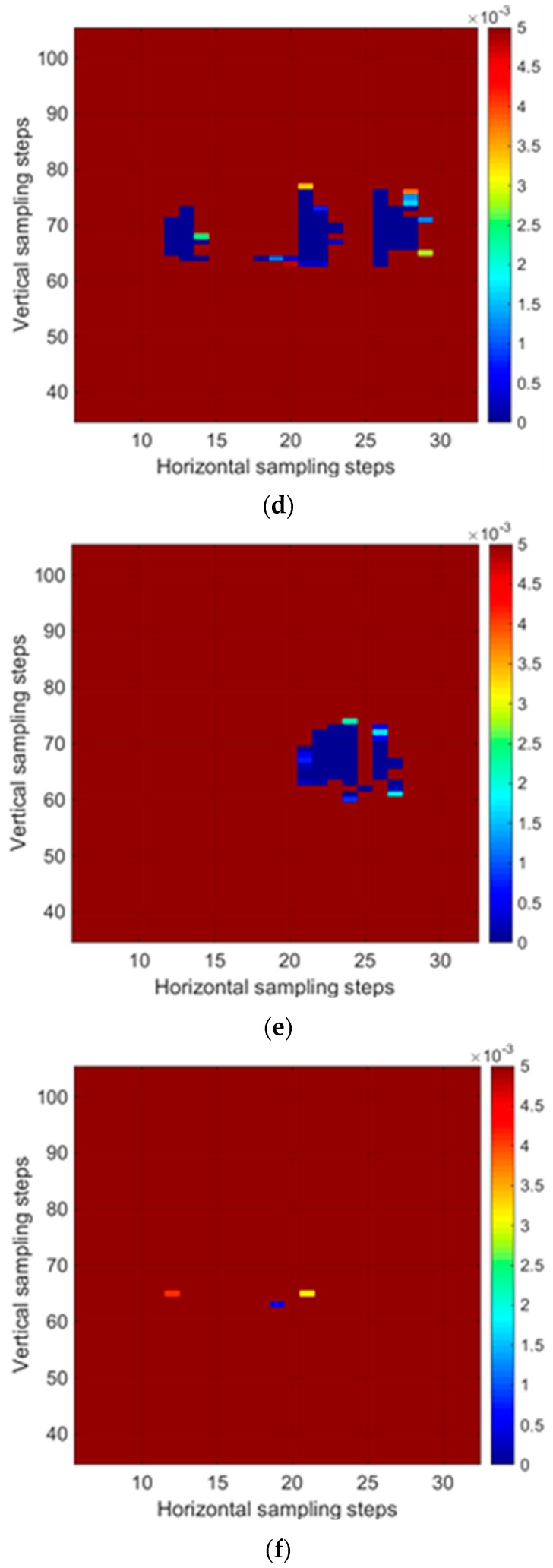
BER matrix during the test times (Test #1) of (**a**) 37, (**b**) 850, (**c**) 1684, (**d**) 1713, (**e**) 1745, and (**f**) 1766 min.

Figure 14 shows the evolution of the BER matrix from Test #1. While the eye size gradually decreased until around 1684 min, i.e., the time to failure, it began to lose its shape thereafter due to continued chemical stress. These results indicate that the BER matrix reflects SI, and has the potential to serve as an on-chip interconnect health sensing method in real time without requiring additional sensing devices.

## 5. Conclusions

In this paper, we presented an on-chip health sensing method for non-invasive and non-destructive interconnect degradation monitoring. Digital signals were transmitted and received through an interconnect of interest. Using SI analyses, it was demonstrated that the BER matrix can be used to detect interconnect faults without requiring additional sensing devices, as early as DC resistance did under accelerated life testing. The eye size of the BER matrix showed the highest separation between normal and abnormal states, indicating its potential for serving as an interconnect health indicator.

These experimental results imply that interconnect fault sensing can be performed without requiring additional sensing devices, and thus, without interrupting system operation. Thus, the use of this on-chip health sensing method for critical interconnects or components should allow for electronics-based system health monitoring in real time and avoiding failure by providing early warnings of impending failure. These warnings can provide a basis for system diagnosis and prognosis, and, in turn, effective system health management.

The proposed on-chip health sensing method can eventually substitute current DC resistance-based health sensing by embedding it into electronics-based systems. No requirement of external and additional sensing devices should allow users to have real-time access to system health status, providing users with opportunity for system diagnosis and prognosis for proactive asset management. This data-based decision-making practice should be able to reduce potential system reliability concern, increasing system availability.

However, the proposed on-chip health sensing method has limitations in terms of measurement methodology and conditions. Because the proposed method measures the BER using digital signals, the components of interest should be in operation and transmitting digital signals. Moreover, a dedicated sensing circuitry, including a transmitter and receiver, would be needed within the electrical circuit to construct a signal transmission channel. Thus, failure modes, mechanisms, and effect analysis (FMMEA) would be helpful to prioritize the components to be monitored when implementing our proposed method.

Our future work on this subject will involve additional acceleration testing under different stress conditions, use of combined features, and statistical anomaly detection techniques for automatic fault diagnosis, such as sequential probability ratio test (SPRT) and statistical process control (SPC) charts.

## Figures and Tables

**Table 1:** Feature set description. BER—bit error rate.

No.	Feature	Description
A1	Eye width	Number of sampling time steps where BER is lower than 10^−17^
A2	Eye height	Number of threshold voltage steps where BER is lower than 10^−17^
A3	Eye size	Number of BER sampling points where BER is lower than 10^−17^
A4	BER average	Average of BER matrix

**Table 2:** Separation analysis results of the BER features.

Feature	Separation Index			Mean	SD
	Test #1	Test #2	Test #3		
Eye width	7.31	4.28	3.64	5.08	1.96
Eye height	7.59	4.63	3.57	5.26	2.01
Eye size	8.19	5.63	3.78	5.87	2.21
BER average	8.18	3.88	3.25	5.12	2.67

**Table 3:** Time to failure (TTF) based on the measurement features. DC—direct current.

Measurement	TTF (min)		
	Test #1	Test #2	Test #3
DC resistance	1670.7	1668.9	2043.2
BER (eye size)	1680.3	1676.5	2033.8
Relative accuracy	0.0057	0.0046	−0.0046
